# Methanolic Extract of Sambucus ebulus Ameliorates Clinical Symptoms in Experimental Type 1 Diabetes through Anti-Inflammatory and Immunomodulatory Actions

**DOI:** 10.22074/cellj.2021.7287

**Published:** 2021-08-29

**Authors:** Hamid Aghajanzadeh, Mohsen Abdolmaleki, Mohammad Ali Ebrahimzadeh, Nazanin Mojtabavi, Tahereh Mousavi, Maryam Izad

**Affiliations:** 1. Department of Immunology, School of Medicine, Tehran University of Medical Sciences, Tehran, Iran; 2. Razi Vaccine and Serum Research Institute, Karaj, Iran; 3. Pharmaceutical Sciences Research Center, School of Pharmacy, Mazandaran University of Medical Sciences, Sari, Iran; 4. Department of Immunology, School of Medicine, Iran University of Medical Sciences, Tehran, Iran; 5. Multiple Sclerosis Research Center, Tehran University of Medical Sciences, Tehran, Iran

**Keywords:** CD4+ T Cell, CD8+ T Cell, Regulatory T Cell, Sambucu sebulus, Type 1 Diabetes

## Abstract

**Objective:**

*Sambucus ebulus* (SE), a famous traditional Iranian medicine, is grown in the north of Iran. As a traditional medicine with anti-inflammatory effects, SE has been utilized against inflammatory joint diseases, insect bites, infectious wounds, edema, and eczema. Type1 diabetes, is an autoimmune disease, characterized by the destruction of pancreatic beta cells by the immune system. For the first time, we investigated the effect of methanolic extract of SE on CD4+, CD8+ and regulatory T cells in experimental type 1 diabetes (T1D).

**Materials and Methods:**

In this experimental study, fifty-six C57BL\6 mice in 8 groups (G1-G8), were enrolled. Diabetes was induced by a multiple low-dose streptozotocin (MLDS) protocol and mice were daily treated with SE extract at 200 and 400 mg/kg doses, for 35 days. Fasting blood glucose was weekly measured by a glucometer. Islets insulin content was analyzed by immunohistochemistry. Percentage of CD4+, CD8+ and regulatory T cells and cytokines production levels were evaluated by flow cytometer and ELISA, respectively.

**Results:**

The clinical symptoms of diabetes were significantly alleviated in G2 group mice which received 400 mg/ kg SE extract. Immunohistochemistry analysis showed that the insulin content of islets increased in G2 group mice. Immunophenotyping analysis indicated that the percentage of CD4+ and CD8+ T cells in G2 group mice was significantly decreased. SE extract significantly increased the percentage of regulatory T cells. The extract in G2 and G4 groups mice significantly decreased IFN-γ and IL-17levels. The extract significantly increased IL-10 in G2 group mice.

**Conclusion:**

The protective effect of SE extract in MLDS-induced diabetes could be partly due to a decrease of CD4+ and CD8+ T cells and an increase of Treg cells resulting in an inflammation reduction in the pancreatic islets.

## Introduction

Type 1 diabetes (T1D) is the most prevalent chronic metabolic disorder of younger adults in which progressive destruction of insulin-producing β-cells leads to the loss of glucose homeostasis (1). Its prevalence in Iran was estimated to be 11.4% during 2015-2016 (2). Although humoral immunity contributes to the onset of T1D, the disease is generally proposed as a T cell-driven autoimmune disease (1, 3).

Both CD4+ and CD8+ T cells have been considered key effectors involved in T1D development and progression (4). Based on non-obese diabetic (NOD) mice and T1D human studies, dysregulation of homeostasis in cytokines produced by different T cell subsets is believed to induce islet cell inflammation (5). Cytotoxic CD8+ T cells are the prominent T cells infiltrating the islets that mediate direct β-cell destruction through perforin and granzyme B (6). CD8+ T cells producing interferon gamma (IFN-γ) and tumor necrosis factor alpha (TNF-α) as the predominant T cell population infiltrate the islets of patients who died at the onset of T1D (1). Deficient major histocompatibility complex class I (MHC-I) NOD mice are resistant to T1D (7). 

Islet CD4+ T cells destruct β-cells through secretion of pro-inflammatory cytokines (8). T helper 1 (Th1) response can worsen destruction of pancreatic β-cells that leads to hyperglycemia in T1D (9). Th1 cells accelerate T1D progression by producing IFN-γ and interleukin-2 (IL-2), while Th2 cytokines have a protective function in the disease (6, 10). However, Th2 and regulatory T cells (Tregs) have defects in the initiation of T1D so they are unable to control the disease progression (11). The severity of inflammation in the pancreas is closely related to an increased expression of pro-inflammatory cytokines such as IFN-γ and TNF-α and a concomitant reduction in IL-4, IL-5 and IL-10 levels (9). Th17 cells can cause local inflammation and as a result, they can play a role in diabetes development. Inhibition of IL-17 function by therapeutic agents or targeting IL-17-producing cells that contribute to diabetes shows that IL-17 is an important cytokine in T1D pathogenesis (12). Furthermore, a fundamental role has been attributed to Treg cells in the regulation of immune tolerance and the self-reactive response during the progress of this disease (13).

Therapeutic approaches including diet, exercise, transplantation, and pharmaceutical therapy have been used for T1D management (14). Nowadays, traditional herbal medicine is highly regarded as a promising strategy in the management of the disease. Plant-derived agents, with the same efficacy, overcome adverse effects of previous therapies (15). *Sambucus ebulus* (SE), from the Caprifoliaceae family, extensively grows in the northern regions of Iran, has been shown to have anti-inflammatory and anti-nociceptive effects (16). In Iranian traditional medicine, leaves, rhizomes, and roots of the plant have been utilized against inflammatory joint diseases, bee and nettle bites, arthritis and sore throat. Furthermore, SE has been reported to be useful in the treatment of burns and infectious wounds, edema and eczema (17).

The aim of the present research was to examine the anti-diabetic and anti-inflammatory effects as well as CD4+ and CD8+ T cell subsets cytokine pattern following the use of SE leaves extract in streptozotocin (STZ) induced-T1D.

## Materials and Methods

### Preparation of *Sambucus ebulus* extract

SE leaves were collected from Sari, Iran in September 2015. The voucher species of the plant has been deposited in the herbarium of the Faculty of Pharmacy, Sari University of Medical Sciences (herbarium No. 87). The leaves were air-dried at room temperature and then coarsely grounded before extraction. Here, 100 g of sample was fractionated by successive solvent extraction at room temperature using maceration with hexane (250 ml×3) then ethyl acetate (250 ml×3) and finally methanol (250 ml×3), respectively. The methanol extract (the 3^rd^ fraction) was concentrated using a rotary vacuum evaporator (for about 45 minutes at 35˚C) until a solid extract sample was obtained. The crude extract was freeze-dried for complete water removal. The extract was prepared in normal saline and tween 80 (5%) for pharmacological studies and kept at -20˚C until use.

### Animals

In our experimental study, male C57BL/6 mice (age: 6-8 weeks; weight: 18-21 g) were purchased from Pasteur Institute (Tehran, Iran). The mice were housed in cages (7 mice in each cage) with a 12/12 hours light/dark cycle and access to food and tap water in a ventilated room. Temperature and humidity of the room were held at 25 ± 2ºC and 55-60%, respectively. All mice were fed with a pelletized normal commercial chow diet (Orient Bio., Seoul, Republic of Korea) for 7 days after arrival. After one-week adaptation, the animals under went the experiments. The study protocol was approved by the Animal Ethics Committee of Tehran University of Medical Sciences, Tehran, Iran (IR.TUMS.MEDICINE. REC.1398.618).

### Diabetes induction

Experimental autoimmune diabetes was induced using multiple low-dose streptozotocin (MLDS) protocol that closely resembles inflammatory changes within the pancreas in humans. Streptozotocin (STZ, Sigma, USA) was dissolved in 0.1 M citrate buffer (pH=4.5). The freshly prepared STZ was injected within 10 minutes. Mice were injected intraperitoneally (i.p.) with STZ 40 mg/kg daily for 5 consecutive days. Blood glucose levels in mice were measured after 6 hours of fasting. Fasting blood sugar (FBS) concentration was measured in the blood drawn from the tail vein on days 0 (one day before the first dose of STZ), 7, 14, 21, 28, 35 using a glucometer Test-strips (Accu-Check instant, Boehringer Mannheim Corporation, Indianapolis, IN, USA). Mice with FBS over 250 mg/dl were considered diabetic.

### Experimental design

To investigate the efficacy of SE extract in the prevention and treatment of STZ diabetes, animals were randomly divided into 8 experimental groups (7 mice in each group) as follows. G1: mice received both STZ (40 mg/ kg, i.p.) and SE extract (200 mg/kg) simultaneously; G2: mice received both STZ (40 mg/kg, i.p.) and SE extract (400 mg/kg) simultaneously; G3: STZ diabetic mice received 200 mg/kg SE extract 15 days after the first STZ administration; G4: STZ diabetic mice received 400 mg/ kg SE extract 15 days after the first STZ administration; G5 (diabetic control group): mice received both STZ and normal saline containing 5% tween 80 simultaneously; G6(non-diabetic control group): mice received normal saline containing 5% tween 80; G7: healthy mice received 200 mg/kg SE extract and G8: healthy mice received 400 mg/kg SE extract. SE extract was i.p. administered once a day for 35 days (18, 19).

### Splenic cell suspension

Mice were killed by anesthesia using ketamine and xylazine combination. It is 100 mg/kg body weight for Ketamine and 10 mg/kg body weight for Xylazine injected i.p. Single-cells were isolated from the spleens and the erythrocytes were lysed using an isotonic solution of ammonium chloride (pH=7.2). After washing with phosphate-buffered saline (PBS, Euroimmun, Germany), the cell suspensions were used for both phenotypic analysis and cytokine evaluation.

### Lymphocyte immunophenotyping by flowcytometry

Splenocytes suspension was adjusted to 1×10^6 ^cells/100 µl staining buffer. The cells were stained with anti-mouse CD4-PerCP-Cy5.5, CD8-Alexa Fluor 488, CD25- APC,and Foxp3-PE (BioLegend, USA). For the analysis of regulatory CD4+ T cells, the cells were treated by fixation/ permeabilization solution (Biolegend, USA) according to the manufacturer’s instructions. The incubation time was 30 minutes at 4° C. All lymphocytes subsets were analyzed by FACS Calibur instrument (Becton Dickinson, USA). The flow cytometer data analyses were done using the FlowJo software version 7.6.1 (Tree Star, USA).

### Evaluation of Th1, Th2, and Th17 cytokines production

For determination of IFN-γ, IL-10 and IL-17 cytokines level, splenocytes were dispensed in a 24-well flat-bottom tissue culture plate (Orange Scientific, Belgium) at 1×10^6^ cells/ml of RPMI-1640 culture medium (Biosera, France), supplemented with 10% inactivated fetal bovine serum (Gibco, USA100 ,( U/mL penicillin, and 100 mg/ml streptomycin (Biosera, France). The samples were stimulated with Concanavalin A (Con A, Sigma, USA) 10 µg/ml and incubated for 48 hours (37◦ C, 5% CO_2_). The supernatants were then collected and stored at -80◦ C until cytokines measurement. The cytokines were quantified using ELISA kits according to the manufacturer’s instructions (R&D Systems, USA). The lower limit of detection for IFN-γ and IL- 10 were 31.3 pg/ml and for IL-17 was 15.3 pg/ml.

### Immunohistochemistry of pancreas for insulin detection

To investigate the effect of SE extract on insulin secretion by islets cells, we evaluated the insulin content in deparaffinated pancreatic tissue sections using immunohistochemistry. Briefly, the pancreaswas removed and fixed in phosphate buffer containing 10% formalin (pH=7.2). The paraffin blocks were cut into 4-5 µm thick sections. The sections on silane-coated slides (Azma Cell Aria, Iran) were rehydrated and treated with 0.1% hydrogen peroxide (H_2_ O_2_) for 15-20 minutes to block nonspecific background staining. The slides were heated in Tris/EDTA buffer pH=9.0. (Merck, Germany) for 10 minutes and washed three times in PBS. The sections were blocked in PBS buffer containing 0.1% Triton (Merck, Germany) and 1% bovine serum albumin (BSA) for 60 minutes. Then, the slides were incubated with rabbit anti-insulin monoclonal antibody (dilution 1:64000, Abcam, UK) overnight and then with sheep anti-rabbit IgG coupled with peroxidase (dilution 1:300, Ebn-Sina, Iran). Chromogen color development was accomplished with 3, 3-diaminobenzidine tetrahydrochloride (Sigma, USA). The slides were counterstained with Harris’s hematoxylin (Sigma, USA).

### Statistical analysis

Statistical analyses were performed using the SPSS program (SPSS, Chicago, USA). All data are presented as the mean ± SD. Bodyweight and FBS of experimental group sat different time points were analyzed by repeated-measures ANOVA. Comparing CD4+ and CD8+ T cell subsets percentage and cytokines concentration was performed using one-way ANOVA followed by Tukey’s test. P<0.05 were considered to be statistically significant. Furthermore, GraphPad Prism software 5.0 (GraphPad, USA) was used for drawing graphs.

## Results

### Diabetes induction

Diabetes was successfully induced in mice (6-8 weeks old) by STZ injection. FBS concentration in diabetic mice was above 250 mg/dl on days 14 and 15. In diabetic control mice (G5), FBS was significantly higher than healthy controls (G6) (P<0.0001). Mice in the G5 group had polydipsia and polyuria in comparison to the mice of the G6 group. Also, the body weight of the G6 mice was significantly higher than that of the G5 mice (P<0.0001).

### SE extract alleviated clinical symptoms of diabetic mice

**Fig.1:**
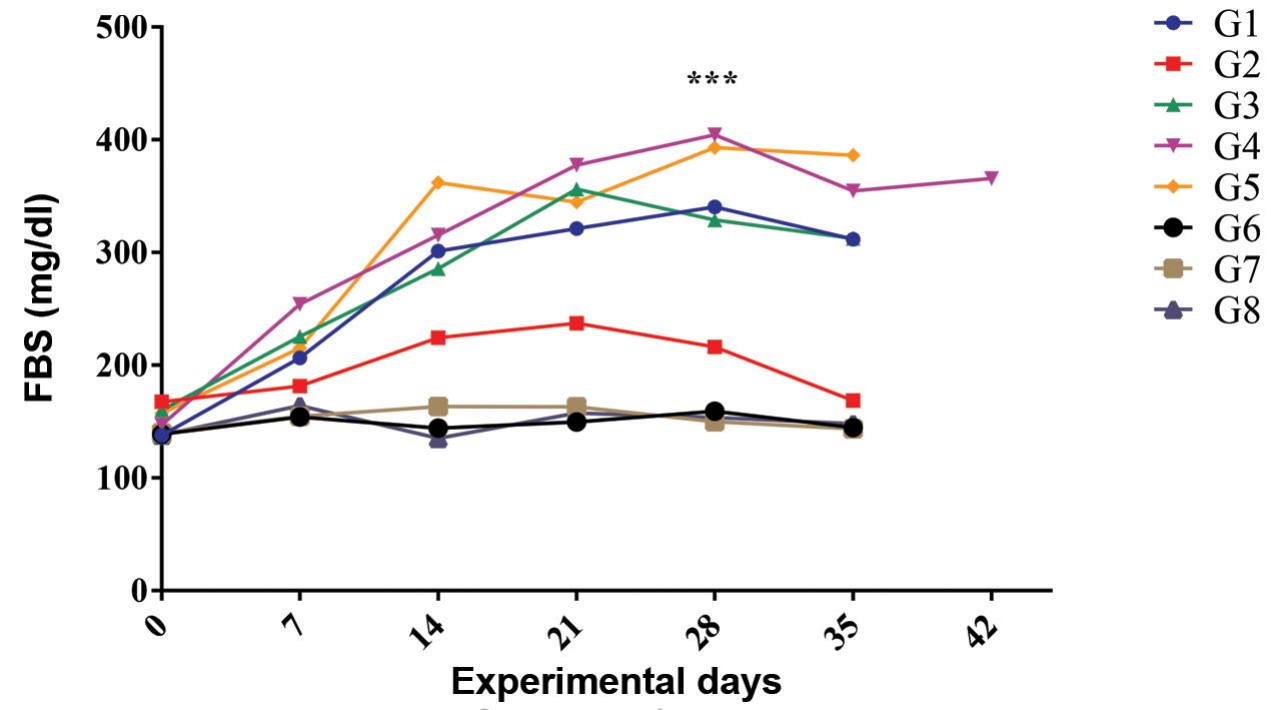
Effect of SE extract on FBS in different experimental groups. To examine the differences between the groups, repeated-measures ANOVA was used and results are shown in a line chart. FBS; Fasting blood sugar, SE; *Sambucus ebulus*, STZ; Streptozotocin, G1; Mice received both STZ (40 mg/kg, i.p.) and SE extract (200 mg/kg) simultaneously, G2; Mice received both STZ (40 mg/kg, i.p.) and SE extract (400 mg/kg) simultaneously, G3; STZ diabetic mice received 200 mg/kg SE extract 15 days after the first STZ administration, G4; STZ diabetic mice received 400 mg/kg SE extract 15 days after the first STZ administration, G5 (diabetic control group); Mice received both STZ and normal saline containing 5% tween 80 simultaneously, G6 (non-diabetic control group); Mice received normal saline containing 5% tween 80, G7; Healthy mice received 200 mg/kg SE extract, G8; Healthy mice received 400 mg/kg SE extract, and ***; P<0.001.

SE extract, at 200 and 400 mg/kg, was administrered simultaneously with diabetes induction (G1 and G2 groups, respectively). The blood glucose levels in diabetic groups started to increase from day day 7. Interestingly, 400 mg/ kg SE extract could significantly prevent the rise of glucose levels in the G2 mice in comparison to the G1 and G5 mice (P<0.0001). There was no significant difference in blood glucose levels in the G3 and G4 mice which received SE both concentrations on day 15, in comparison to the G5 mice. The administration of this extract had no effect on blood glucose levels in non-diabetic mice (the G7 and G8 groups). The blood glucose levels of these mice were similar to those observed in the non-diabetic G6 group which did not receive the extract (Fig .1).

Furthermore, the body weight of the G1, G4, and G5 mice was significantly reduced compared to the G2 and G6 mice (P<0.0001). However, the bodyweight of the healthy groups that only received the extract (the G7 and G8) was not significantly different from that of the G2 and G6 (Table 1). Body weight of the G3 and G4 mice did not differ significantly from the G5 mice.

Mice treated with the 400 mg/kg SE (the G2) did not show polydipsia and polyuria compared to diabetic mice (the G1, G3, G4, and G5), and were similar to non-diabetic mice (the G6, G7, and G8).

### Effect of SE extract on CD4+, CD8+ and regulatory T cells percentage in the spleen of mice 

**Fig.2:**
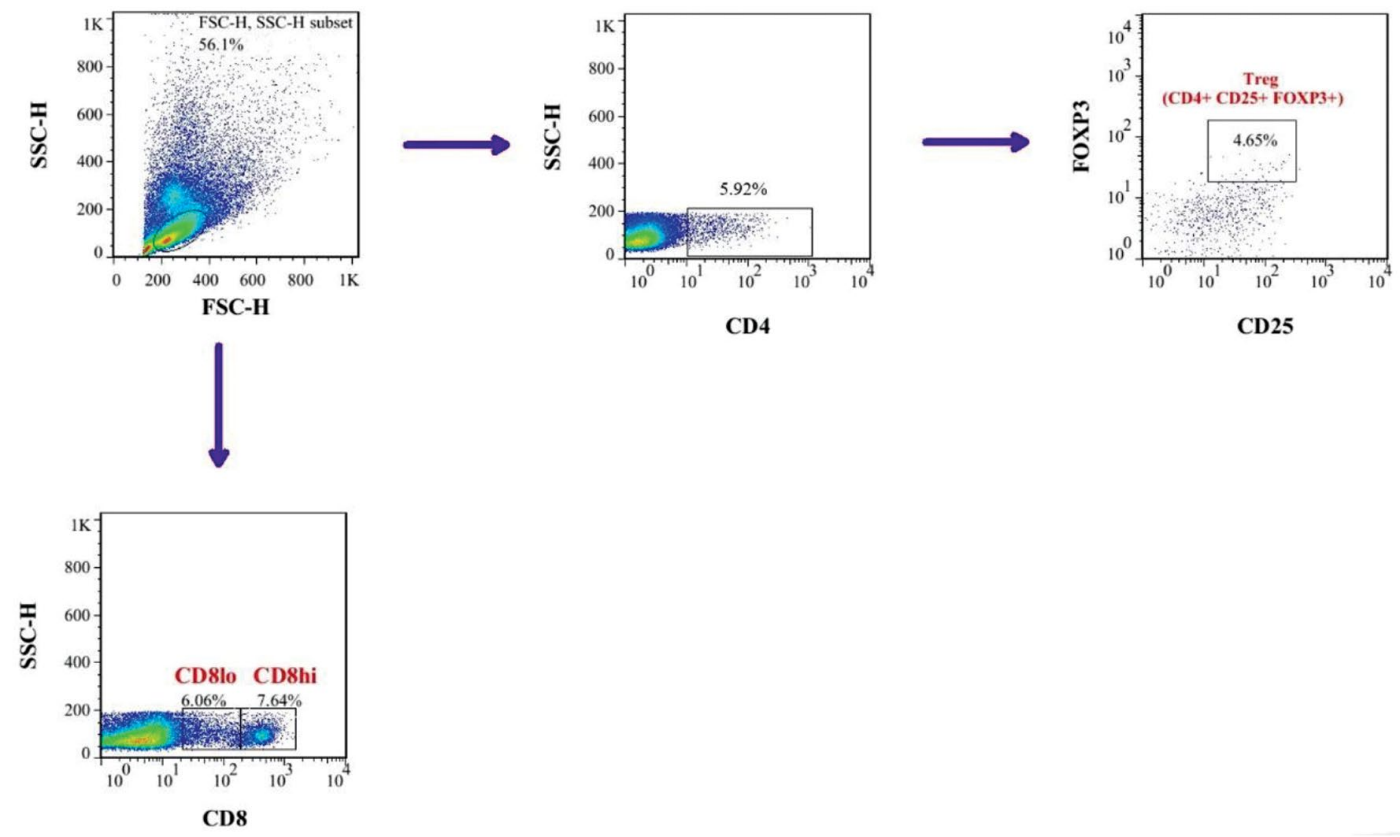
Representative gating strategy for CD4+ and CD8+ cell subsets. Cells were first gated for lymphocytes (SSC-A vs. FSC-A) and CD4+ and CD8+ cells were determined within the indicated gate. The cells were further analyzed for Treg (CD25+ FOXP3+) and CD8 low and CD8hi cells fractions.

To analyze the influence of SE extract on splenic lymphocytes proportions, we assessed the percentage of both CD4+ and CD8+ T cell subsets in the studied experimental groups. First, lymphocytes were gated on a forward vs. side scatter dot plot and then Treg, CD8^low^ and CD8^hi ^cells populations were analyzed on gated cells. At least 50,000 events were counted for each sample. The gating strategy is illustrated in Figure 2.

**Table 1:** Body weight (g) on days 0 and 35 in experimental groups


Group	G1	G2	G3	G4	G5	G6	G7	G8
Day								

0	18.9 ± 1.09	18.9 ± 1.04	19 ± 1.1	18.9 ± 1.11	18.9 ± 1.09	18.6 ± 0.83	19.2 ± 1.42	18.6 ± 1.01
35	15.1 ± 0.66	21.2 ± 1.02	14.6 ± 0.91	15.8 ± 0.57	14.3 ± 0.6	23.6 ± 0.86	24.4 ± 0.81	23.8 ± 0.72
Body weight changes	- 20.1	+12.2	-23.2	-16.4	-24.3	+26.9	+27.1	+28


Data are presented as mean ± SD or %. To examine the differences between G1-G8 experimental groups, repeated-measures ANOVA was used. G1; Mice received both STZ (40 mg/kg, i.p.) and SE extract (200 mg/kg) simultaneously, G2; Mice received both STZ (40 mg/kg, i.p.) and SE extract (400 mg/kg) simultaneously, G3; STZ diabetic mice received 200 mg/kg SE extract 15 days after the first STZ administration, G4; STZ diabetic mice received 400 mg/ kg SE extract 15 days after the first STZ administration, G5 (diabetic control group); Mice received both STZ and normal saline containing 5% tween 80 simultaneously, G6 (non-diabetic control group); Mice received normal saline containing 5% tween 80, G7; Healthy mice received 200 mg/kg SE extract, G8; Healthy mice received 400 mg/kg SE extract, SE; *Sambucus ebulus*, and STZ; Streptozotocin.

**Fig.3:**
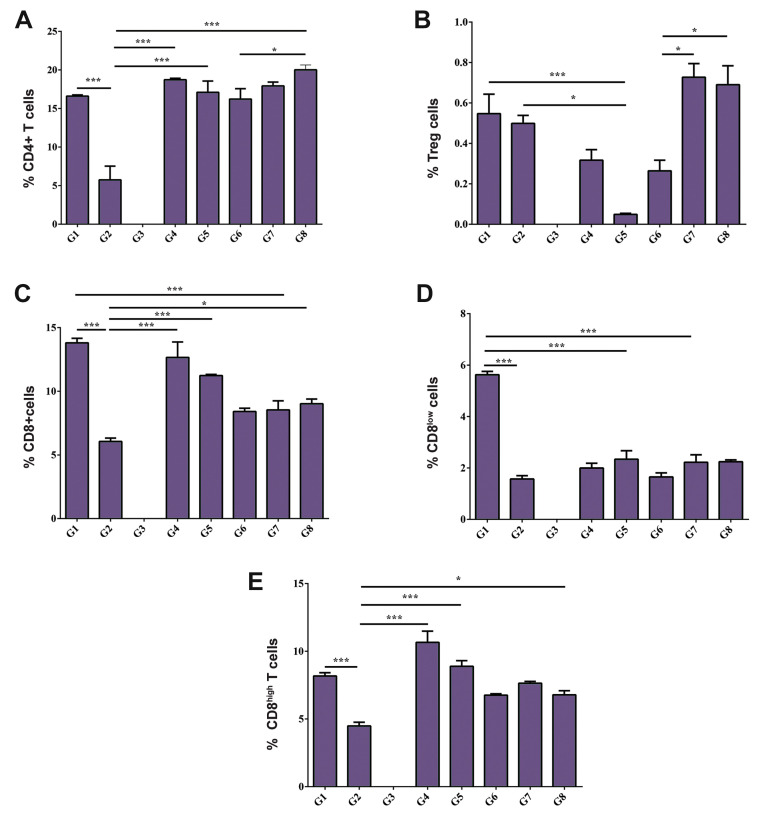
CD4+, CD8+ and Treg cell percentage in different experimental groups. The percentage of **A.** CD4+, **B.** Treg, **C.** CD8+, **D. **CD8^low^, and **E.** CD8^hi^ cells in G1-G8 experimental groups. One-way ANOVA was used to test for differences among the groups. Tukey’s test was performed for subsequent pairwise comparison. G1; Mice received both STZ (40 mg/kg, i.p.) and SE extract (200 mg/kg) simultaneously, G2; Mice received both STZ (40 mg/kg, i.p.) and SE extract (400 mg/kg) simultaneously, G3; STZ diabetic mice received 200 mg/kg SE extract 15 days after the first STZ administration, G4; STZ diabetic mice received 400 mg/kg SE extract 15 days after the first STZ administration, G5 (diabetic control group); Mice received both STZ and normal saline containing 5% tween 80 simultaneously, G6 (non-diabetic control group); Mice received normal saline containing 5% tween 80, G7; Healthy mice received 200 mg/kg SE extract, G8; Healthy mice received 400 mg/kg SE extract, *; P<0.05, ***; P<0.001, SE; *Sambucus ebulus*, and STZ; Streptozotocin.

We observed that 400 mg/kg SE extract promoted a significant increase in CD4+ cells in the G8 as compared to the G6 (P<0.05, Fig .3A). The SE extract at both 200 and 400 mg/kg significantly augmented the percentage of Treg cells in the G1 and G2 groups respectively, in comparison to the G5 mice (P<0.0001 and P<0.01, respectively, Fig .3B). The percentage of Treg cells in the G2 mice in comparison with the G1 mice was not significantly different. Notably, the percentage of Treg cells in healthy mice receiving the extract (the G7 and G8) was significantly increased compared to the healthy control mice (the G6) (P<0.01, Fig .3B).

However, SE extract at 400 mg/kg (the G2) significantly decreased CD4+, CD8+ and CD8^high^ lymphocytes percentage in comparison to diabetic mice (the G1, G4 and G5) (P<0.0001), whereas CD8^low^ cells reduced in the G2 compared to the G1 (Fig.3A, C, D, E, respectively).

### Effect of SE extract on IFN-γ, IL-17, and IL-10 cytokines production

**Fig.4:**
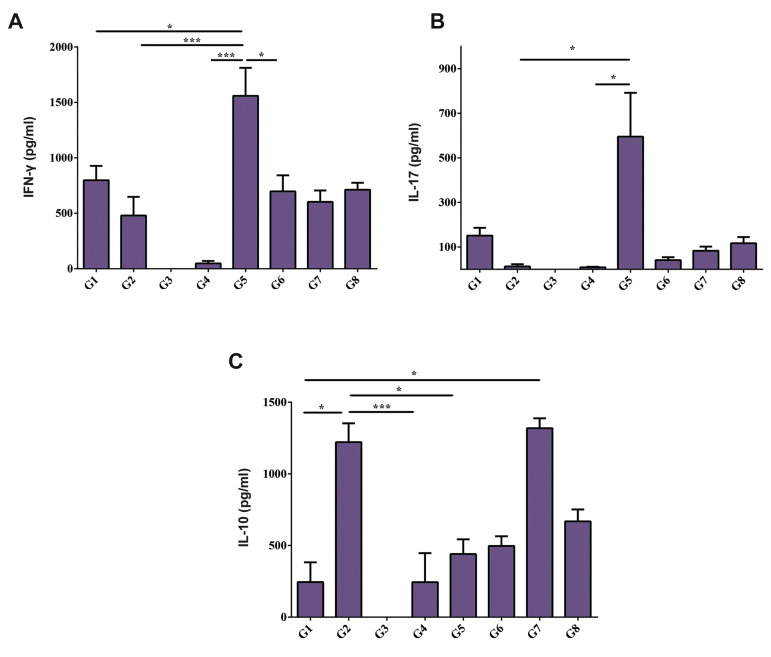
The levels of IFN-γ, IL-17, and IL-10 in the supernatant of cultured splenocytes. Comparison of **A. **IFN-γ, **B. **IL-17, **C.** IL-10 cytokines concentrations among G1-G8 experimental groups. One-way ANOVA followed by a subsequent Tukey’s multiple pairwise comparison test was done to examine the differences between the groups. IFN- γ; Interferon gamma, IL; Interleukin, G1; Mice received both STZ (40 mg/kg, i.p.) and SE extract (200 mg/kg) simultaneously, G2; Mice received both STZ (40 mg/kg, i.p.) and SE extract (400 mg/kg) simultaneously, G3; STZ diabetic mice received 200 mg/kg SE extract 15 days after the first STZ administration, G4; STZ diabetic mice received 400 mg/kg SE extract 15 days after the first STZ administration, G5 (diabetic control group); Mice received both STZ and normal saline containing 5% tween 80 simultaneously, G6(non-diabetic control group); Mice received normal saline containing 5% tween 80, G7; Healthy mice received 200 mg/kg SE extract, and G8; Healthy mice received 400 mg/kg SE extract, *; P<0.05, and ***; P<0.001.

Our data revealed that the diabetic control group (the G5) had a significantly higher IFN-γ level than the non-diabetic control group (the G6) (P<0.05, Fig .4A). Besides, 400 mg/kg dose of SE extract significantly prevented the rise of IFN-γ and IL-17 in the G2 and G4 compared to the diabetic control group (the G5) (P<0.001 and P<0.05, respectively, Fig .4A, B). Interestingly, the concentration of IL-10 increased in the supernatant of splenocytes culture of G2mice compared to diabetic mice (the G1, G4 and G5) (Fig .4C). We however observed a lower concentration of IFN-γ in mice treated with 200 mg/kg SE extract (the G1) compared to the control diabetic mice (the G5) (P<0.05, Fig .4A).

### Effect of SE extract on insulin production in pancreatic islets

**Fig.5:**
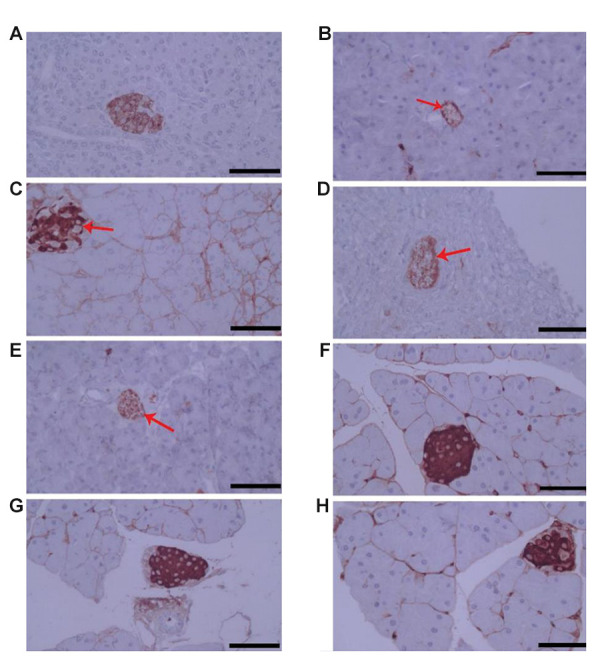
Insulin production in pancreatic islets of mice that received SE extract (scale bar: 100 μm). **A. **Positive control group, **B. **G1: received STZ and 200 mg/kg SE extract simultaneously, **C. **G2: received STZ and 400 mg/kg SE extract simultaneously, **D.** G4: received 400 mg/kg SE extract after induction of diabetes on day 15, **E.** G5: received STZ and normal saline containing 5% tween 80 (diabetic control), **F. **G6: received normal saline containing 5% tween 80 (non-diabetic control), **G. **G7: received only 200 mg/kg SE extract (non-diabetic mice), and **H.** G8: received only 400 mg/kg SE extract (non-diabetic mice). The arrows show pancreatic islets.

Immunohistochemistry (IHC) analysis of pancreatic tissues in different groups of mice showed increased production of insulin in the G2 mice but a significant reduction in the G1, G4 and G5 groups. The amount of insulin production in healthy mice receiving the extract (the G7and G8) was similar to that of healthy control mice (the G6) (Fig .5).

## Discussion

T1D is a chronic autoimmune disease that occurs in genetically susceptible individuals (7). The disease begins with destruction of the pancreatic beta cells because of autoimmune Th1, TCD8+, and macrophage attacks,resulting in decreased insulin production and increased blood glucose levels (1). Currently, T1D treatment mainly involves insulin use. The main disadvantage of this therapeutic approach is having no effect on the autoimmune process of the disease, which is a key event in the pathogenesis of T1D (15). There are various immunotherapies that reduce lymphocyte subsets, induce immune tolerance and activate or increase Treg cells (14). In various studies, SE has been shown as an antioxidant, anti-inflammatory and immunomodulatory agent. Among the various extracts of this plant, its methanolic extract has the best anti-inflammatory activity based on previously published papers (14, 17).

For the first time, we showed that the SE extract was able to prevent the development of experimentaldiabetes so that after 35 days of extract injection, FBS in diabetic mice reached the normal range. Releasing of autoantigens results in immune cell responses, inflammation in the pancreas and increased blood glucose within 2 weeks (20, 21). 

In the G2 mice, the FBS level slowly increased from day 7 to 21 and then decreased significantly and reached the normal range on day 35. However, blood glucose levels in the G6, G7 and G8 mice were normal. Previously, reduction of blood glucose levels was shown following the use of the extracts of Red ginseng, *Uncaria tomentosa, Cassia auriculata and Bauhinia forficate* (10, 22). Additionally, we and others showed that the anti-inflammatory effects of the herbals were dose-dependent (10). It seems that 400 mg/kg SE increases the survival of mice (mice in the G4 group vs G3 survived until the end of the study although their blood glucose was high). This protective effect can be linked to various mechanisms, such as restoring pancreatic function (14), regeneration of beta cells (14) and increasing insulin production (15). 

FBS level did not change in the G7 and G8 mice respectively receiving 200 and 400 mg/kg of the extract compared to the G6 mice. Therefore, it can be said that the extract had no effect on glucose metabolism. This effect was also shown following the use of the methanolic extract of *Origanum vulgare* (23).

Immunohistochemistry analysis of pancreatic tissues has shown that 400 mg/kg of the extract increased insulin production (G2) due to an increase in the number of healthy islets in the pancreas in comparison with the diabetic control group (the G5), whereas insulin production significantly reduced in the G1, G4 and G5 mice in which a large number of pancreatic islets was damaged by STZ, compared to the G6 mice. Similar findings were obtained in previous studies using *Uncaria tomentosa and Origanum vulgare* extracts (10, 23). Since insulin production in the pancreas of the G7 and G8 mice was similar to the G6, we conclude that the extract does not directly affect insulin metabolism.

Both CD8+ and CD4+ T cells are involved in T1D pathogenesis and can cause the death of beta cells (24). Direct destruction of beta cells is accomplished by cytotoxic T lymphocyte cells that can recognize MHC I and auto-antigens complexes on beta cells (6). NOD mice deficient in MHC I were resistant towards against T1D. CD8+ T cells have been found in the peripheral blood of T1D patients. These cells, both in the early stages of diabetes and in its final phase, can damage the pancreatic islets (25). Th1 cells may have different roles in the development of T1D by producing IFN-y and IL-2. IFN-γ plays a role in the destruction of beta-cells via the STAT-1 pathway.IL-2 can be effective in treating T1D by increasing the survival rate and function of Treg cells (6). In this study, we observed that 400 mg/kg of the extract could decrease the percentages of CD4+ and CD8+ including CD8^hi ^and CD8^low^ T cells in the G2 mice. CD8+ T cells are divided into two groups based on the level of the expression of CD8: CD8^high^ and CD8^low^ T cells (26). High levels of perforin and granzyme B in CD8^low^ T cells indicate their role as cytotoxic/effector T cells. These cells also produce significant amounts of IFN-γ (27). Therefore, these cells can be harmful to beta cells. On the other hand, the two doses of SE decreased IFN-γ production by the G1, G2 and G4 mice compared to the G5 animals, which could be due to the reduction in the percentage of TCD4+ and TCD8+ cells, the main cytokine producers. In this study, IFN-γ was increased in the G5 mice compared with the G6 animals, whereas the other two cytokines (IL-10 and IL-17) were not significantly different which indicates an increase in the number or activity of CD8+ T cells or in the polarization of Th cells toward Th1. In another study, diabetic mice produced more IFN-γ than normal mice, but no differences were observed in IL-4 levels (10).

In the development of T1D, a defect in the function of Treg cells may be more important than a defect in their number (6). Patients with T1D and NOD mice have a relative deficiency in Treg number and function. In the absence of Treg cells, the onset of T1D is accelerated and the severity of the disease increases, which can indicate the important role of these cells in T1D (13). Both doses of SE increased the percentage of Treg cells in mice of the G1 and G2 groups compared to the G5 mice. IL-10, a cytokine produced by Tregs, only increased in the G2 mice compared to the G5 mice. Although 200mg/kg of SE in the G1 mice significantly increased the percentage of Treg cells, it did not control FBS and other symptoms of diabetes, while 400 mg/kg of SE in theG2 mice significantly increased both the Treg number and IL-10 level, controlled diabetes symptoms and reduced FBS. Thus the plant extract may have an effect on the function of Treg cells, rather than on their number. A significant reduction in the percentage of Treg cells was also shown in STZ-induced diabetic mice while, the use of *Uncaria tomentosa *extract reach the percentage of Treg cells to the percentage level of healthy mice (10).

Th17 cells play an important role in T1D by increasing the destruction of islets via Th1 cells. Th17 cells in the T1D pathogenesis can also stimulate TCD8 + cells (25). Th2 cells produce IL-10, a cytokine that regulates immunity and plays an important role in providing immune tolerance in NOD mice (1). Inflammatory cytokines IL-17 and IFN-γ were reduced in the G1, G2 and G4 mice and anti-inflammatory cytokine IL-10 only increased in the G2 mice. Therefore, the protective effect of the SE extract may be partly related to the immunomodulatory effect of the Th2 cytokine profile. This extract may have triggered the polarization of Th cells from Th1 to Th2 or from Th17 to Treg or a combination of both. In other studies, the use of *Uncaria tomentosa *extract increased IL-4 and IL-5 levels, suggesting that the cytokine profile could be adjusted to Th2 (10). Oral administration of a parasitic fungus called *Cordyceps sinensis* significantly delayed the onset of T1D and reduced its severity. This effect is attributed to an increase in the ratio of Treg to Th17 cells (25). It was also found that *Origanum vulgare* extract altered the signaling pathway of Pro-Th17 to Pro-Treg, and the number of Th2 and Treg cells in mice treated with this extract was higher than that of diabetic mice (23).

The extract of SE also has antioxidant properties (16, 17). Since macrophages and their products from the reactive oxygen species (ROS) and reactive nitrogen species (RNS) pathways can induce destruction of pancreatic beta cells, this extract is likely to prevent beta-cell apoptosis by neutralizing these products and helpin improving pancreatic conditions for insulin production. In a similar study, *Origanum vulgare* extract was able to neutralize ROS and RNS production and prevent beta-cell apoptosis (23).

## Conclusion

Overall, it is probable that the protective effect of SE extract in MLDS-induced diabetes is at least partly due to a reduction in the process of inflammation in the pancreatic islets. These results indicate that SE can protect mice against autoimmune diabetes and in the future, it can be valuable in treating diabetes and other autoimmune and inflammatory diseases in humans. Of course, more research is needed on this subject.

## References

[1] van Belle TL, Coppieters KT, von Herrath MG (2011). Type 1 diabetes: etiology, immunology, and therapeutic strategies. Physiol Rev.

[2] Esteghamati A, Larijani B, Haji Aghajani M, Ghaemi F, Kermanchi J, Shahrami A (2017). Diabetes in Iran: prospective analysis from first nationwide diabetes report of national program for prevention and control of diabetes (NPPCD-2016). Sci Rep.

[3] Knip M, Siljander H, Ilonen J, Simell O, Veijola R (2016). Role of humoral beta-cell autoimmunity in type 1 diabetes. Pediatr Diabetes.

[4] Kent SC, Babon JAB (2017). Narrowing in on the anti-β cell-specific T cells: looking ‘where the action is’. Curr Opin Endocrinol Diabetes Obes.

[5] He JS, Xie PS, Luo DS, Sun CJ, Zhang YG, Liu FX (2014). Role of immune dysfunction in the pathogenesis of type 1 diabetes mellitus in children. Asian Pac J Trop Med.

[6] Li M, Song LJ, Qin XY (2014). Advances in the cellular immunological pathogenesis of type 1 diabetes. J Cell Mol Med.

[7] Eringsmark Regnell S, Lernmark A (2013). The environment and the origins of islet autoimmunity and Type 1 diabetes. Diabet Med.

[8] Fatima N, Faisal SM, Zubair S, Ajmal M, Siddiqui SS, Moin S (2016). Role of pro-inflammatory cytokines and biochemical markers in the pathogenesis of type 1 diabetes: correlation with age and glycemic condition in diabetic human subjects. PLoS One.

[9] Singh B, Nikoopour E, Huszarik K, Elliott JF, Jevnikar AM (2011). Immunomodulation and regeneration of islet beta cells by cytokines in autoimmune type 1 diabetes. J Interferon Cytokine Res.

[10] Domingues A, Sartori A, Golim MA, Valente LMM, da Rosa LC, Ishikawa LLW (2011). Prevention of experimental diabetes by Uncaria tomentosa extract: Th2 polarization, regulatory T cell preservation or both?. J Ethnopharmacol.

[11] Russell MA, Morgan NG (2014). The impact of anti-inflammatory cytokines on the pancreatic β-cell. Islets.

[12] Martin-Orozco N, Chung Y, Chang SH, Wang YH, Dong C (2009). Th17 cells promote pancreatic inflammation but only induce diabetes efficiently in lymphopenic hosts after conversion into Th1 cells. Eur J Immunol.

[13] Brusko T, Atkinson M (2007). Treg in type 1 diabetes. Cell Biochem Biophys.

[14] Schneider DA, Kretowicz AM, von Herrath MG (2013). Emerging immune therapies in type 1 diabetes and pancreatic islet transplantation. Diabetes Obes Metab.

[15] Greenbaum C, Lord S, VanBuecken D (2017). Emerging concepts on disease-modifying therapies in type 1 diabetes. Curr Diab Rep.

[16] Jabbari M, Daneshfard B, Emtiazy M, Khiveh A, Hashempur MH (2017). Biological effects and clinical applications of dwarf elder (Sambucus ebulus L): a review. J Evid Based Complementary Altern Med.

[17] Ahmadiani A, Fereidoni M, Semnanian S, Kamalinejad M, Saremi S (1998). Antinociceptive and anti-inflammatory effects of Sambucus ebulus rhizome extract in rats. J Ethnopharmacol.

[18] Mahmoudi M, Ebrahimzadeh MA, Dooshan A, Arimi A, Ghasemi N, Fathiazad F (2014). Antidepressant activities of Sambucus ebulus and Sambucus nigra. Eur Rev Med Pharmacol Sci.

[19] Fathi H, Ebrahimzadeh MA, Ziar A, Mohammadi H (2015). Oxidative damage induced by retching; the antiemetic and neuroprotective role of Sambucus ebulus L. Cell Biol Toxicol.

[20] Arora S, Kumar Ojha S, Vohora D (2009). Characterization of Streptozotocin induced diabetes mellitus in swiss albino mice. Glob J Pharmacol.

[21] Kleinert M, Clemmensen C, Hofmann SM, Moore MC, Renner S, Woods SC (2018). Animal models of obesity and diabetes mellitus. Nat Rev Endocrinol.

[22] Hong YJ, Kim N, Lee K, Sonn CH, Lee JE, Kim ST (2012). Korean red ginseng (Panax ginseng) ameliorates type 1 diabetes and restores immune cell compartments. J Ethnopharmacol.

[23] Vujicic M, Nikolic I, Kontogianni VG, Saksida T, Charisiadis P, Orescanin-Dusic Z (2015). Methanolic extract of Origanum vulgare ameliorates type 1 diabetes through antioxidant, anti-inflammatory, and anti-apoptotic activity. Br J Nutr.

[24] Khadra A, Pietropaolo M, Nepom GT, Sherman A (2011). Investigating the role of T-cell avidity and killing efficacy in relation to type 1 diabetes prediction. PLoS One.

[25] Shao S, He F, Yang Y, Yuan G, Zhang M, Yu X (2012). Th17 cells in type 1 diabetes. Cell Immunol.

[26] Izad M, Harirchian MH, Amiri H, Najafi F, Ghaflati Z, Salehi Z (2013). Low and high CD8 positive T cells in multiple sclerosis patients. Iran J Allergy Asthma Immunol.

[27] Trautmann A, Ruckert B, Schmid-Grendelmeier P, Niederer E, Brocker EB, Blaser K (2003). Human CD8 T cells of the peripheral blood contain a low CD8 expressing cytotoxic/effector subpopulation. Immunology.

